# pH-Sensitive Dendrimersomes of Hybrid Triazine-Carbosilane Dendritic Amphiphiles-Smart Vehicles for Drug Delivery

**DOI:** 10.3390/nano10101899

**Published:** 2020-09-23

**Authors:** Evgeny Apartsin, Nadezhda Knauer, Valeria Arkhipova, Ekaterina Pashkina, Alina Aktanova, Julia Poletaeva, Javier Sánchez-Nieves, Francisco Javier de la Mata, Rafael Gómez

**Affiliations:** 1 Institute of Chemical Biology and Fundamental Medicine SB RAS, 8, Lavrentiev ave., 630090 Novosibirsk, Russia; knauern@gmail.com (N.K.); v.arkhipova@g.nsu.ru (V.A.); pashkina.e.a@yandex.ru (E.P.); aktanova_al@mail.ru (A.A.); fabaceae@yandex.ru (J.P.); 2 Department of Natural Sciences, Novosibirsk State University, 630090 Novosibirsk, Russia; 3 Laboratoire de Chimie de Coordination, CNRS, 31077 Toulouse, France; 4 Research Institute of Fundamental and Clinical Immunology, 630099 Novosibirsk, Russia; 5 Departamento de Química Orgánica y Química Inorgánica, UAH-IQAR, Universidad de Alcalá, 28805 Alcalá de Henares, Spain; javier.sancheznieves@uah.es (J.S.-N.); javier.delamata@uah.es (F.J.d.l.M.); 6 Networking Research Center on Bioengineering, Biomaterials and Nanomedicine (CIBER-BBN), 28029 Madrid, Spain; 7 Instituto Ramón y Cajal de Investigación Sanitaria, IRYCIS, 28034 Madrid, Spain

**Keywords:** carbosilane, dendron, triazine, amphiphile, self-assembly, vesicles, pH-sensitive, doxorubicin, methotrexate, leukaemia

## Abstract

Supramolecular constructions of amphiphilic dendritic molecules are promising vehicles for anti-cancer drug delivery due to the flexibility of their architecture, high drug loading capacity and avoiding off-target effects of a drug. Herein, we report a new class of amphiphilic dendritic species—triazine-carbosilane dendrons readily self-assembling into pH-sensitive dendrimersomes. The dendrimersomes efficiently encapsulate anticancer drugs doxorubicin and methotrexate. Chemodrug-loaded dendrimersomes have dose-related cytotoxic activity against leukaemia cell lines 1301 and K562. Our findings suggest that triazine-carbosilane dendrimersomes are prospective drug carriers for anti-cancer therapy.

## 1. Introduction

The use of engineered nanosized constructions as drug delivery systems has become an emerging area of attention in recent years due to their ability to improve pharmacological properties of cargo compounds, i.e., improving selectivity and efficiency of drug delivery into target cells/tissues, reducing off-target effects of drugs, and protecting drugs from the biological environment amid the delivery [1]. Furthermore, nanoconstructions are expected to release a cargo in a controllable manner upon the action of external stimuli (changes of ambient pH or temperature, enzymatic digestion etc.) [2]. In light of this, the use of supramolecular assemblies for drug delivery seems to be very promising [3].

Dendritic molecules, dendrimers and dendrons, are versatile platforms to design supramolecular nanoconstructions for robust drug delivery [4,5,6,7,8]. In particular, carbosilane dendrimers are efficient tools for antibacterial [9] and antiviral therapy [10], nucleic acid delivery [11], cancer treatment [12], imaging [13], functional nanomaterials [14,15], and so on. It should be noted that dendritic architectures give room to design various types of supramolecular constructions based on the molecular topology of building blocks. Systematic studies conducted using amphiphilic dendrons revealed the effect of both the structure of the hydrophobic part and the dendron generation as amphiphilic part on their assembly into supramolecular constructions of given topology: micelles, unilamellar or multilamellar vesicles [16,17,18]. These regularities are implemented in the development of dendron-based nanoconstructions for biomedical applications [19,20,21,22,23].

To increase the loading capacity, the formation of vesicle-like assemblies (dendrimersomes) is preferred over micelle-like ones. This can be achieved by branching the hydrophobic part in the focal point of dendron. In this work, we suggest the use of a triazine moiety as a branching point. Triazine-based synthons can be easily prepared by the controllable substitution in cyanuric chloride [24]; they have already been shown to be versatile building blocks both for building dendrimer scaffolds [25] and for the functionalisation of dendrimer surface [26]. Furthermore, triazine cycle gets protonated in slightly acidic medium (pH~5.5) [27], which can be useful for the design of pH-sensitive constructions [28].

Herein, we report the synthesis of a new class of functional dendritic species—amphiphilic triazine-carbosilane dendrons. We aim to study the self-assembly of dendrons in physiological conditions as well as to explore the potential of dendrons’ self-assemblies for drug delivery. As a representative and emerging model, leukaemia cell lines have been chosen. Leukaemia is a severe cancer of frequent occurrence. At present, despite advances of chemotherapy, the survival rate in adult patients with acute lymphoblastic leukaemia does not exceed 50% [29]. The prognosis for chronic leukaemia is more favorable; however, some aggravations, so-called blast crises that require special treatment may occur [30]. In view of this, the development of novel approaches to increase the efficacy of chemotherapy, in particular, nanomedicine-based ones, is highly important.

## 2. Materials and Methods

### 2.1. General Information

Organic solvents were dried and freshly distilled under argon prior to use. Reagents were obtained from commercial sources and used as received. Dendron precursor BrG_2_V_4_ was obtained as described elsewhere [31].

Water solutions of chemicals, amphilhiles, and chemodrugs as well as buffer solutions were prepared using milliQ^®^ deionized water. Sonication of amphiphile solutions was done in a Sonorex Super RK 31 H ultrasonic bath (Bandelin Electronic, Berlin, Germany). Dialysis was done using SnakeSkin dialysis membranes MWCO 3500 (Thermo Fisher, Waltham, MA, USA).

### 2.2. Analytical and Spectroscopic Techniques

^1^H and ^13^C and spectra were recorded on Varian Unity VXR-300 (Varian Inc., Palo Alto, CA) and Bruker AV400 (Bruker, Karlsruhe, Germany) instruments. Chemical shifts (δ, ppm) were measured relative to residual ^1^H and ^13^C resonances for CDCl_3_ used as solvent. ESI-TOF analysis was carried out in an Agilent 6210 TOF LC/MS mass spectrometer (Agilent, Santa Clara, CA, USA).

UV-vis spectra were recorded using an Eppendorf Biospectrophotometer (Eppendorf, Hamburg, Germany). Measurements were done at 25 °C using 1 mm thick quartz cells. Fluorescence spectra were recorded using a CLARIOstar microplate reader (BMG LABTECH, Ortenberg, Germany).

Hydrodynamic diameter of the supramolecular aggregates obtained was determined in plastic disposable microvolume cells using a Zetasizer Nano ZS particle analyzer (Malvern Instruments, Manchester, UK), equipped with NBS. The measurements were made at 25 °C. Zeta potential values were measured in plastic disposable cells DTS 1061 using Malvern Instruments Nanosizer ZS particle analyser. 10 mM of Na phosphate buffer was used to prepare solutions.

Transmission electron microscopy (TEM) images were obtained using a Veleta digital camera (EM SIS, Muenster, Germany) mounted on a JEM 1400 transmission electron microscope (JEOL, Tokyo, Japan) at the accelerating voltage of 80 kV. Samples were stained with 0.1% uranyl acetate.

### 2.3. Cell Experiments

Human T-cell leukemia cell line 1301 and human chronic myelogenous leukemia cell line K562 (European collection of authenticated cell cultures, Sigma Aldrich, Merck KGaA, Germany) were used. Cells were cultivated in the RPMI-1640 cell medium containing 10% foetal calf serum (HyClone Laboratories, South Logan, UT, USA), 0.3 mg/mL L-glutamine (Vector, Russia), 50 µg/mL gentamicin (DalChimPharm, Khabarovsk, Russia), and 25 µg/mL tienam (Merck Sharp & Dohme, Kenilworth, NJ, USA) in a humidified atmosphere containing 5% CO_2_ at 37 °C.

### 2.4. Statistical Analysis

We used GraphPad Prism software and Statistica 7.0 software for data analysis and visualization. The Mann–Whitney criterion was used; the differences were considered to be significant if *p* < 0.05.

### 2.5. Synthesis of Dendron Amphiphiles

#### 2.5.1. 2,4-dodecylamino-6-chloro-1,3,5-triazine

Cyanuric chloride (970 mg, 5.26 mmol) and dodecylamine (2.0 g, 10.8 mmol) were mixed in 150 mL CHCl_3_. The mixture was cooled with an ice bath, then 10% aqueous KOH was added up to the pH~10. The reaction mixture was stirred overnight at room temperature, then organic phase was separated, the solvent was evaporated under vacuum, and the solid residue was recrystallized from the mixture CHCl_3_:CH_3_OH (5:1) to yield the desired product as white solid (2.28 g, 90%).


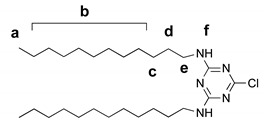


^1^H NMR (400 MHz, CDCl_3_) δ 0.86 (t, J = 6.8 Hz, 6H, H_a_), 1.18–1.37 (m, 32H, H_b_), 1.52–1.64 (m, 8H, H_c_, H_d_), 3.45 (q, J = 6.8 Hz, 4H, H_e_), 6.03 (s, 2H, H_f_). ^13^C NMR (101 MHz, CDCl_3_) δ 14.1, 22.6, 26.6, 28.5–29.9 (m), 31.9, 41.5, 165.8, 169.7, 171.0. MS: [M + H]^+^ 482.40 amu (calcd 481.39 amu).

#### 2.5.2. 2,4-dodecylamino-6-piperazino-1,3,5-triazine

2,4-didodecylamino-6-chloro-1,3,5-triazine (600 mg, 1.2 mmol) and piperazine (645 mg, 7.5 mmol) were mixed in 15 mL CHCl_3_; the reaction mixture was stirred at room temperature. When the starting triazine derivative was fully consumed, as shown by TLC (5% CH_3_OH in CH_2_Cl_2_), the solution was washed several times with 1M NaOH, then with water. The organic phase was separated, dried over Na_2_SO_4_, and the solvent was removed under vacuum to yield the desired product as yellowish solid (600 mg, 92%).


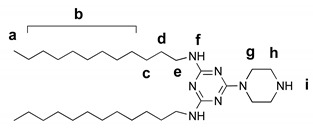


^1^H NMR (400 MHz, CDCl_3_) δ 0.87 (t, J = 6.5 Hz, 6H, H_a_), 1.20–1.37 (m, 36H, H_b_, H_c_), 1.52 (t, 4H_d_), 1.81 (s, 1H, H_i_), 2.85 (s, 4H, H_h_), 3.33 (d, 4H, H_g_), 3.72 (s, 4H, H_e_), 4.75 (s, 2H, H_f_). ^13^C NMR (101 MHz, CDCl_3_) δ 14.1, 22.7, 27.0, 28.6–30.3 (m), 31.9, 40.7, 44.2, 46.1, 165.2, 166.3. MS: [M + H]^+^ 532.51 amu (calcd 531.50 amu).

#### 2.5.3. Vinyl-Terminated Dendron G2

2,4-didodecylamino-6-piperazino-1,3,5-triazine (220 mg, 0.41 mmol) and vinyl-terminated carbosilane dendron BrG_2_V_4_ (140 mg, 0.3 mmol), K_2_CO_3_ (70 mg, 0.5 mmol) were mixed in 30 mL acetone in a sealed ampule with catalytic amounts of 18-crown-6 and KI added. The reaction mixture was stirred for 24 h at 90 °C. The reaction completion was monitored by TOCSY ^1^H NMR following the disappearance of BrCH_2_ protons. When the reaction was over, the solvent was removed under vacuum, the residue was dissolved in ethyl acetate and washed with brine. The organic phase was dried over Na_2_SO_4_, and the solvent was removed. The crude product was purified by silica gel column chromatography (eluent: ethyl acetate:hexane 1:1) to yield functionalized dendron as light-yellow oil (245 mg, 90%).


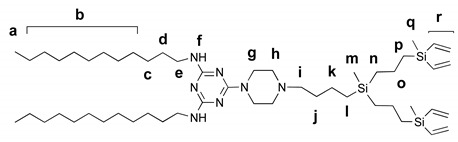


^1^H NMR (400 MHz, CDCl_3_) δ –0.12 (s, 3H, H_m_), 0.10 (s, 6H, H_q_), 0.46 (m, 2H, H_l_), 0.53 (m, 4H, H_n_), 0.67 (m, 4H, H_p_), 0.84 (t, J = 6.5 Hz, 6H, H_a_), 1.19–1.35 (m, 42H, H_b_, H_c_, H_k_, H_o_), 1.50 (t, J = 7.3 Hz, 6H, H_d_, H_j_), 2.32 (t, J = 7.8 Hz, 2H, H_i_), 2.42 (t, J = 5.1 Hz, 4H, H_h_), 3.30 (d, J = 6.6 Hz, 4H, H_g_), 3.76 (s, 4H, H_e_), 4.88 (s, 2H, H_f_), 5.59–6.21 (m, 12H, H_r_). ^13^C NMR (101 MHz, CDCl_3_) δ -5.2, -5.1, 13.9, 14.1, 18.3, 18.5, 18.7, 22.0, 22.7, 26.9, 28.7–30.1 (m), 30.7, 30.9, 31.9, 40.6, 42.9, 53.2, 58.6, 132.6, 137.1, 164.7, 166.1.

#### 2.5.4. Amphiphilic Dendron G2

Functionalized vinyl-terminated dendron (400 mg, 0.44 mmol), 2-(dimethylamino)ethanethiol hydrochloride (275 mg, 1.76 mmol) and dimethoxyphenylacetophenone (DMPA) (12 mg, 0.044 mmol) were dissolved in 5 mL of mixture THF:CH_3_OH (1:2). The reaction mixture was deoxygenated by bubbling argon and irradiated by UV for 2 h (365 nm, 120W). Then, another 12 mg (0.044 mmol) of DMPA was added, and the reaction mixture was irradiated for another 2 h. The reaction completion was monitored by ^1^H NMR. After the reaction was completed, the solvents were removed under vacuum and then the residue was dissolved in methanol. Afterward, it was precipitated in diethyl ether, and after the solvent was separated, the solid was dried under vacuum to afford the desired dendron as light-yellow solid (495 mg, 75%). For characterization, the dendron was deprotonated with K_2_CO_3_.


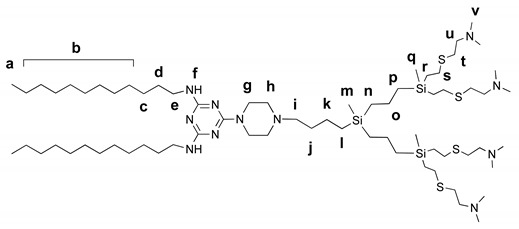


^1^H NMR (400 MHz, CDCl_3_) δ −0.10 (s, 3H, H_m_), −0.01 (s, 6H, H_q_), 0.46 (m, 2H, H_l_), 0.52 (m, 4H, H_n_), 0.59 (m, 4H, H_p_), 0.85 (s, 6H, H_a_), 1.25 (m, 42H, H_b_, H_c_, H_k_, H_o_), 1.49 (m, 6H, H_d_, H_j_), 2.23 (s, 24H, H_v_), 2.32 (m, 2H, H_i_), 2.40 (m, 4H, H_h_), 2.47 (m, 8H, H_u_), 2.53 (m, 8H, H_s_), 2.61 (m, 8H, H_t_), 3.31 (s, 4H, H_g_), 3.75 (s, 4H, H_e_), 4.71 (s, 2H, H_f_). ^13^C NMR (101 MHz, CDCl_3_) δ -5.3, -5.1, 14.1, 14.6, 18.3, 18.7, 22.1, 22.7, 27.0, 27.7, 29.0–30.1 (m), 31.9, 37.0, 40.6, 42.9, 45.4, 53.3, 59.3, 127.7, 128.5, 165.0, 166.2. MS: [M + H]^+^ 1329.00 amu (calcd 1327.99 amu).

### 2.6. CMC Measurements

Association of dendron molecules into supramolecular constructions was studied by fluorescence spectroscopy using pyrene as a marker [32]. Aliquots of pyrene solution in acetone (5 µL, 20 µM) were dispensed in 200 µL glass vials and the solvent was removed under vacuum. Then, solutions of the amphiphilic dendron (100 µL, 0.3 to 100 µM) in 10 mM sodium phosphate buffer, pH 7.0 (PB) or 10 mM phosphate-buffered saline, pH 7.0–7.4 (PBS) were added. Samples were sonicated for 30 min and then incubated for 2 days at room temperature. After the incubation, the samples were transferred into the wells of 96-well black microplate COSTAR 96 half-area, and pyrene fluorescence spectra were recorded in the range 360–500 nm upon excitation at 345 ± 5 nm. The ratio of fluorescence intensities I_373_/I_383_ was plotted against −lgC, fitted with Boltzmann sigmoidal curve (r^2^ > 0.95), and the −lg(CMC) values were estimated from the fitting data.

### 2.7. Study of the pH-Dependence of the Particle Size

The water solution of amphiphilic dendron in a glass vial (1 mL, 100 µM) was sonicated for 30 min and then incubated overnight at room temperature. 100 µL aliquots of the solution were taken into plastic Eppendorf-type test tubes, and 5 µL of 200 mM sodium phosphate buffer (pH 7.0; 6.5; 6.0; 5.5; 5.0) was added. The samples were incubated at room temperature for 2 h, then DLS profiles of the samples were recorded.

### 2.8. Drug Encapsulation

A drop of water solution of amphiphilic dendron (10 µL, 10 mM) and a drop of water solution (10 µL, 10 mM) of a doxorubicin hydrochloride (DOX), sodium methotrexate (MTX) or 5-fluorouracil (5FU) were rapidly mixed in 1 mL of deionized water; an UV-vis spectrum of the sample **[As-mixed]** was recorded. The solution was sonicated for 30 min and then incubated for 2 days at room temperature; an UV-vis spectrum of the sample **[After treatment]** was recorded. Non-encapsulated chemodrug was removed by dialysis against deionized water (4 × 200 mL, 6 h); an UV-vis spectrum of the sample **[After dialysis]** was recorded. The degree of chemodrug encapsulation was estimated from UV-vis spectra as a ratio of a chemodrug absorption at λ_max_ before and after dialysis. Drug loading capacity was estimated as a ratio of the weight of encapsulated drug to the total weight of nanoparticles neglecting the weight of the dendron washed off during the dialysis. The real loading capacity values are thus higher than estimated.

**Figure 1:**
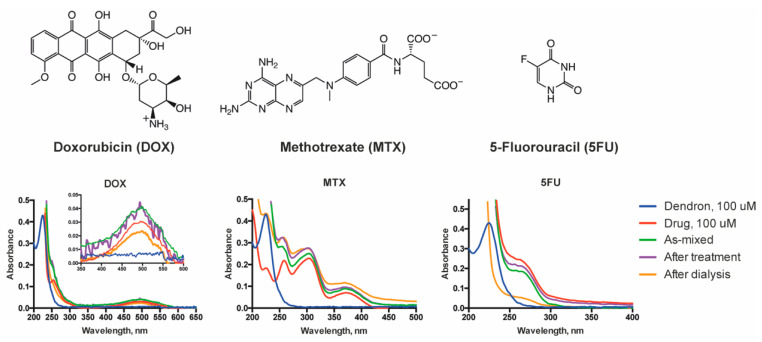
Structures of the chemodrugs used in the work: doxorubicin (DOX), methotrexate (MTX) and 5-fluorouracil (5FU) (**top**). UV-vis spectra of chemodrugs upon encapsulation into dendrimersomes (**bottom**).

The UV-vis spectra of chemodrugs at different stages of encapsulation into dendrimersomes are shown in the Figure 1. DLS and zeta potential distribution profiles of drug-loaded dendrimersomes were recorded.

### 2.9. Drug Release from Dendrimersomes

Doxorubicin-loaded dendrimersomes solution (65 µM DOX) was placed in a 200 µL Eppendorf-type plastic tube, sealed with a dialysis membrane and incubated in 5 mL of 10 mM Na phosphate buffer pH 7.0; 6.5; 6.0 for 24 h upon gentle stirring. At given time points, 50 µL aliquots of dialysis buffer were taken, and their fluorescence was read at 590 nm. Fluorescence intensity was plotted vs. incubation time and fitted with a first order kinetic curve (r^2^ > 0.95).

### 2.10. WST Assay

10^4^ 1301 cells were cultivated in total volume 100 µL in flat-bottomed 96-well cell culture plates (TPP, Switzerland) for 72 h in the presence of blank chemodrug-loaded dendrimersomes as well as free drugs. Non-treated cells were cultivated in parallel as a control.

After cultivation, 10 µL of the WST-1 reagent (Takara Bio Inc, Kusatsu, Japan) per well was added, and the plates were incubated for 4 h. To evaluate the cell viability, optical absorbance at 450 nm was directly read against the background control, the reference was read at 620 nm (TriStar LB 941 Multimode Microplate Reader, Berthold Technologies GmbH&Co., Bad Wildbad, Germany). Cell viability was calculated as a ratio of absorbance of treated cells samples to that of non-treated control, then converted into percentage. Cell viability values were plotted against lgC, fitted with Boltzmann sigmoidal curve (r^2^ > 0.95), and the −lg(IC_50_) values were estimated from the fitting data.

### 2.11. Drug Internalization Studies

0.5 × 10^6^ 1301 cells were cultivated in total volume 100 µL in flat-bottomed 48-well cell culture plates (TPP, Switzerland) for 4 h. Then, water solutions of amphiphilic dendron (5 µM), doxorubicin (3 µM) or doxorubicin-loaded dendrimersomes (doxorubicin content 3 µM) were added. The equal volume of PBS was added to control cells. After cultivation, cells were collected, washed once with PBS, then 50 µL of acidic glycine solution were added, gently pipetted for 30 s and washed again with PBS. Flow cytometric analysis was performed on a FACSCanto II cytometer (BD Biosciences, San Jose, CA, USA). The analysis was made in FITC and PE channels. 15,000 to 50,000 events per sample were acquired and analysed using FACSDiva 6.1.2 software.

1301 cells were incubated with blank dendrimersomes, doxorubicin and doxorubicin-loaded dendrimersomes (6.6 µM DOX) in complete cell culture media for 4 h in humidified atmosphere containing 5% CO_2_ at 37 °C, then fixed in ethanol and glacial acetic acid. Samples were stained with DAPI solution (1.5 µg/mL) with the addition of antifade solution (10 µL) for 20 min, protected from light. The cells were visualized under fluorescence microscopy (Axiopscop 40, Zeiss, Oberkochen, Germany).

### 2.12. Apoptosis Induction Studies 

0.5 × 10^6^ 1301 cells were cultivated in total volume 250 µL for 72 h with amphiphilic dendron (5 µM), doxorubicin (3 µM) or doxorubicin-loaded dendrimersomes (doxorubicin content 3 µM). Non-treated cells were cultivated in parallel as a control. After cultivation, cells were collected, washed twice in cold Cell Staining Buffer (Biolegend, San Diego, CA, USA) and resuspended in Annexin V Binding Buffer (Biolegend, CA, USA). Ice-cold 70% ethanol solution was used for the necrosis induction control; and doxorubicin-treated cells were taken as the apoptosis induction control. For cell staining, the solutions of FITC Annexin V (5 µL per probe with 0.25–1.0 × 10^7^ cells) and 7-AAD (5 µL per probe with 0.25–1.0 × 10^7^ cells) were added. Cells were gently pipetted and incubated for 15 min at room temperature in the dark. Then, 400 µL of Annexin V Binding Buffer were added to each tube. Flow cytometric analysis was performed on a FACSCanto II cytometer (BD Biosciences, San Jose, CA, USA). 40,000 to 100,000 events per sample were acquired and analyzed using FACSDiva software. All experiments were run in triplicates.

## 3. Results and Discussion

### 3.1. Synthesis of the Amphiphilic Dendron

The novel dendritic amphiphile designed herein consists of a branched hydrophobic moiety (substituted triazine) and a cationic carbosilane dendron conjugated through a piperazine moiety, a convenient linker allowing further dendron grafting in relatively mild conditions.

**Scheme 1:**
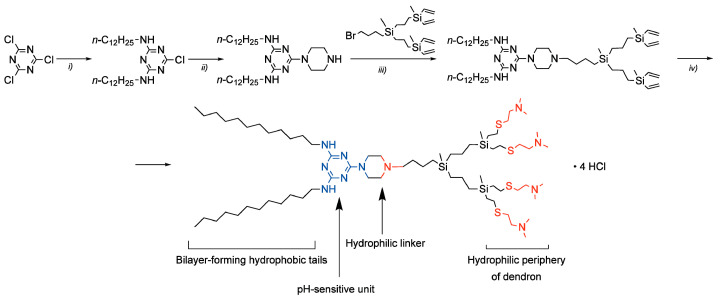
Synthesis of the amphiphilic dendron: (**i**) *n*-C_12_H_25_NH_2_, CHCl_3_, NaOH (aq.), 90%; (**ii**) piperazine, CHCl_3_, 92%; (**iii**) K_2_CO_3_, 18-crown-6, KI, acetone, 90%; (**iv**) HS(CH_2_)_2_N(CH_3_)_2_·HCl, DMPA, 365 nm UV, THF:CH_3_OH, 75%.

We have synthesized the amphiphile in a convergent way (Scheme 1). The hydrophobic triazine unit has been obtained by the substitution of two chlorides in cyanuric chloride with dodecylamino-residues followed by the subsequent grafting of piperazine. The vinyl-terminated carbosilane dendron G2 having bromine in the focal point has been obtained starting from 4-bromobutylmethyldichlorosilane by iterative hydrosilylation and Grignard reaction steps as described earlier [33]. Two units, namely triazine and dendron ones, were conjugated by simple nucleophilic substitution, and vinyl residues at the periphery of the dendron have been modified via thiol-ene reaction. The resulting amphiphilic molecule contains two hydrophobic tails long enough to form a bilayer, a stimuli-sensitive triazine unit, and a branched polycationic dendron unit.

### 3.2. Self-Assembly of Dendrimersomes

**Figure 2:**
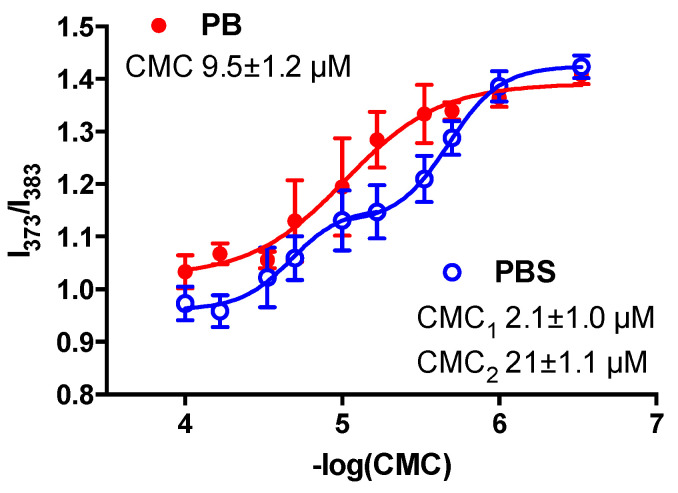
I_373_/I_383_ plots corresponding to the amphiphilic dendron association in PB and PBS.

Dendritic amphiphile molecules are efficiently self-organised into supramolecular associates in water medium. The CMC determination plots in PB and PBS are given in the Figure 2. Comparing two plots, a clear difference is observed. In a low-salt solution (PB), there is only one association stage, quite broad in terms of concentration range, with CMC of 9.5 ± 1.2 µM. In contrast, at physiological salt concentration (PBS), there are two association stages with CMC of 2.1 ± 1.0 µM and 21 ± 1.1 µM. Apparently, the high salt concentration fosters the association of dendron molecules at low concentrations, with these aggregates further rearranging into vesicle-like dendrimersomes at higher concentrations.

**Figure 3:**
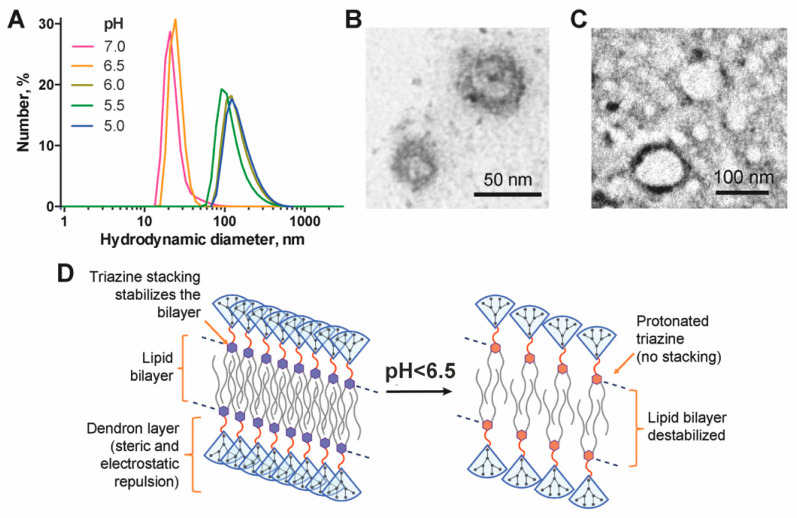
Self-assembly of dendritic amphiphiles. DLS profiles for supramolecular associates of dendron (100 µM) exposed to different pH in 10 mM Na-phosphate buffer (**A**); representative TEM images of dendrons’ supramolecular associates at pH 7.0 (**B**) and pH 5.0 (**C**); tentative scheme of the pH-dependent behaviour of a dendron bilayer (**D**).

Vesicle-like nanoconstructions, dendrimersomes, are readily assembled upon quite simple treatment (short sonication and relaxation). High uniformity of nanoconstructions’ topology and size, likely defined by the molecular structure of the dendron precursor, is achieved without specific post-treatment (e.g., extrusion). Having been formed at neutral pH, dendrimersomes have mean diameter of 20–30 nm; however, when put into acidic medium (pH below 6.5), they reorganize into larger particles 100–150 nm diameter (Figure 3). TEM shows that the vesicle-like structure of dendrimersomes is maintained upon reorganization.

Based on the findings above, we suggest a provisional scheme of the bilayer behaviour (Figure 3D). The main driving force of self-assembly is the interaction of hydrophobic tails of amphiphilic dendron molecules additionally stabilized by stacking interactions of triazine moieties [34]. On the other hand, the bilayer structure is destabilized by the steric and electrostatic repulsion of dendron fragments. Such a balance of factors favouring and unfavouring the bilayer stability likely gives the supramolecular flexibility to the whole dendrimersome. In acidic medium, triazine moieties get protonated, and the stacking is distorted. Thus, the stabilizing factor turns into the destabilizing one; this causes loosening of the bilayer followed by fusion with other vesicles.

It is worth noting that the effects of pH-sensitivity are observed at slightly acidic pH (below 6.5), which corresponds to early stages of endosome maturation. Thus, once delivered into a cell by endocytosis, an encapsulated biologically active cargo can be released into the cytosol in the most favourable moment to escape endosomes. Fusing dendrimersomes can contribute to the endosomal escape release by assisting the endosome membrane disruption.

### 3.3. Drug Loading into Dendrimersomes

**Figure 4:**
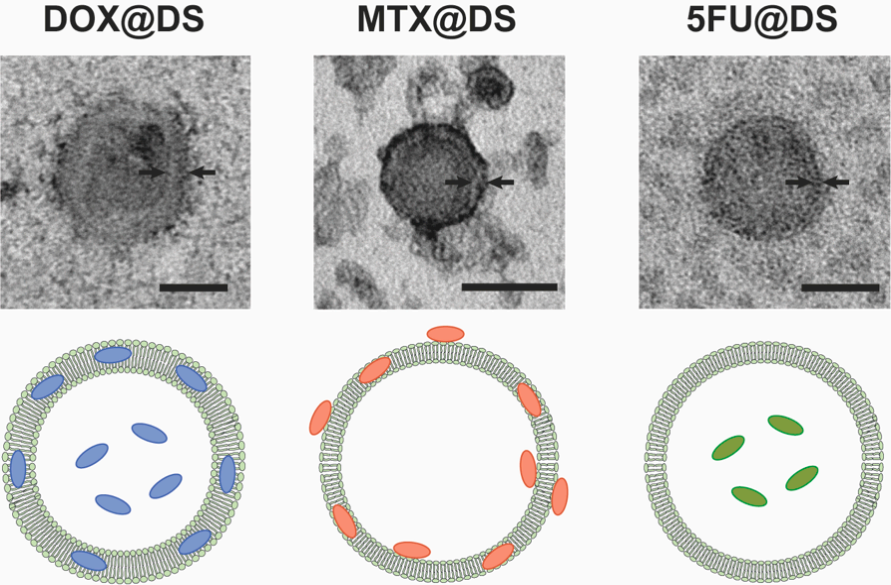
Representative TEM images of doxorubicin-loaded (DOX@DS), methotrexate-loaded (MTX@DS) and 5-fluorouracil-loaded (5FU@DS) dendrimersomes (top) and cartoons showing tentative drug distribution in dendrimersomes (bottom). Scale bar represents 50 nm. Black arrows show the thickness of a bilayer.

To explore the potential of dendrimersomes as drug carriers, we encapsulated three anti-tumor drugs—doxorubicin, methotrexate and 5-fluorouracil into dendrimersomes. Concentrated solutions of a drug and a dendron were rapidly mixed in water, sonicated and incubated for 48 h, followed by removal of non-encapsulated drug by dialysis. This procedure yields drug-loaded dendrimersomes with low size dispersion (PDI < 0.3) with a high degree of drug encapsulation (ca. 65% for doxorubicin and 75% for methotrexate) (Table 1). On the contrary, 5-fluorouracil encapsulated much less efficiently: for ca. 25%. The differences in the drug encapsulation efficiency resulted in 10-fold variation of the drug loading capacity: ~20% for doxorubicin and methotrexate vs. ~2% for 5-fluorouracil (Table 1). Such a dissimilarity is likely explained by the hydrophobicity of the encapsulated compound: doxorubicin and methotrexate can not only be entrapped into the inner cavity of a dendrimersomes but also incorporated into a bilayer (that can be observed in the TEM images (Figure 4) by the thickening of dendrimersomes’ membranes), whereas for 5-fluorouracil, the entrapment likely prevails. Thus, the efficiency of 5-fluorouracil encapsulation can give an idea of the efficiency of the physical entrapment of compounds into the hydrophilic interior of dendrimersomes. It should be noted that methotrexate appeared to be additionally retained on the dendrimersomes’ surface due to electrostatic interactions, as suggested by zeta potential values and TEM images. Drug-loaded dendrimersomes have mean diameter less than 100 nm that makes them suitable for drug delivery studies (Table 1).

We have studied the pH dependence of the drug release from dendrimersomes using doxorubicin-loaded constructions as a model. The release was followed by measuring fluorescence intensity of dialysate (see Section 2.9). The rate of the drug release from dendrimersomes has not been found to increase significantly in slightly acidic medium (Figure S1): The half-release time being 6.3 ± 1.4 h at the pH 7.0 decreased to 6.0 ± 1.5 h (pH 6.5) and 5.4 ± 1.4 h (pH 6.0). However, overall fluorescence intensity of dialysate was higher with the decrease of the pH. The completeness of the doxorubicin release from dendrimersomes after 24 h was >80%. Thus, the pH-dependent behaviour shown for blank dendrimersomes (Figure 3) likely takes place in drug-loaded constructions as well, triggering the release of a drug in slightly acidic media frequently occurring in endocytic vesicles at early stages of endosome maturation [35].

### 3.4. Cytotoxic Activity of Drug-Loaded Dendrimersomes Towards Cancer Cells

We have tested the performance of triazine-carbosilane dendrimersomes as drug carriers by doing a comparative study of cytotoxic effects of nanoconstructions loaded with doxorubicin and methotrexate towards two leukaemia cell lines, 1301 and K562. 5-Fluorouracil-loaded dendrimersomes were excluded from the study due to the insufficient encapsulation efficiency resulting in the low concentration of the drug in the nanoparticle preparations.

Cell lines 1301 and K562 have been chosen as targets, for they represent leukaemia variants related to different types of progenitor cells: 1301 is the acute T-cell leukaemia cell line; K562 is chronic myelogenous leukaemia cell line derived from the cells of a patient having blast crises [36]. Thus, the use of these cell lines permits to estimate the efficiency of drug-loaded nanoconstructions in the haemoblastosis therapy.

**Figure 5:**
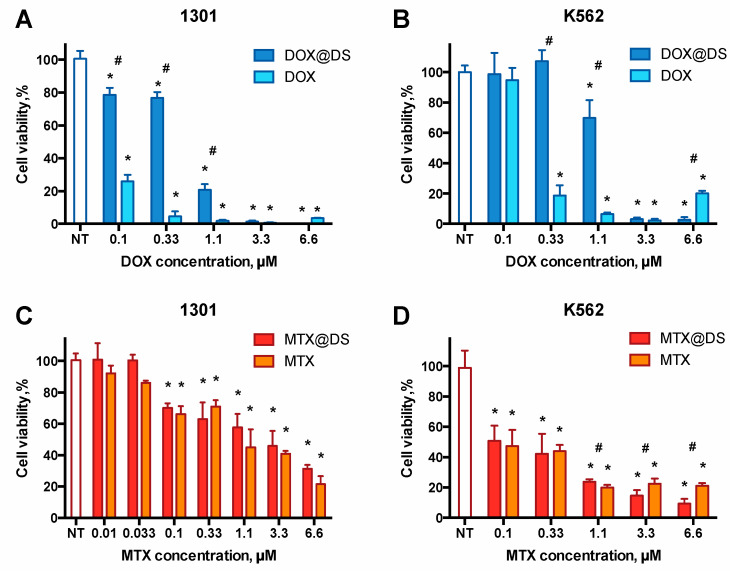
Viability profiles of 1301 (**A**,**C**) and K562 (**B**,**D**) cells after incubation with free chemodrugs and drug-loaded dendrimersomes. NT—non-treated cells. Incubation for 72 h, then WST-1 assay, data are presented as Mean ± S.D. (n = 5). * *p* < 0.05 vs. NT; ^#^ *p* < 0.05 free drug vs. drug@DS.

The ability of drug-loaded dendrimersomes to suppress the viability of target cell lines was studied using WST-1 assay. Dose-response cytotoxic effects of nanoconstructions were observed. Doxorubicin-loaded dendrimersomes have been found to be less cytotoxic in comparison with free drug at low concentrations (below 1 µM) and exhibit similar activity at higher concentrations (Figure 5A,B). These differences are likely connected with the slow release of doxorubicin from dendrimersomes, so, being taken at low concentrations, they do not cause a commeasurable cytotoxic effect, which results in higher cell viability after treatment. In the case of methotrexate, free drug and drug-loaded dendrimersomes possess similar cytotoxicity (Figure 5C,D): In most cases, there were no statistically significant differences found between two groups. In general, the cytotoxic effects have been observed at dendron concentrations below the IC_50_ (Table 2, Figure S2), which permits to assign them to the encapsulated chemodrugs. 

The differences observed between effects of doxorubicin- and methotrexate-loaded dendrimersomes on the viability of target cells are likely explained by the distribution of drugs in dendrimersomes. Since doxorubicin is mostly retained in the hydrophobic part of a vesicle, it is released as a result of reorganization (or even dissociation) of the carrier nanoparticle. Meanwhile, methotrexate is retained mostly at the surface of dendrimersomes, so it can be released relatively easier.

To understand the effects of drug-loaded dendrimersomes better, we have quantified cell subpopulations at different stages of cell death after the treatment with blank dendrimersomes, free drug and drug-loaded nanoconstructions. As a model system, we have taken doxorubicin-loaded dendrimersomes and 1301 cells as a cell model.

**Figure 6:**
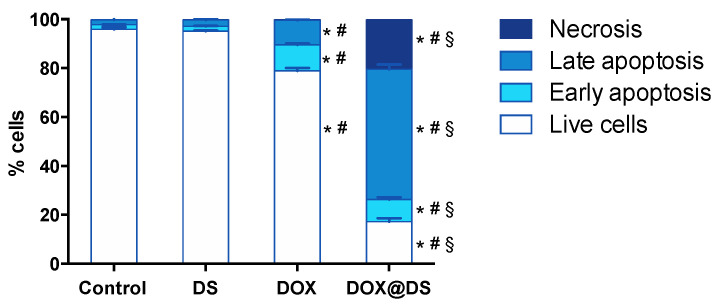
Induction of cell death in 1301 cells by blank dendrimersomes (DS), free doxorubicin (DOX) and doxorubicin-loaded dendrimersomes (DOX@DS). Incubation for 72 h, then FITC-Annexin V/7AAD staining, flow cytometry. Data are presented as mean ± S.D. (n = 3). * *p* < 0.05 vs. control group; ^#^ *p* < 0.05 vs. DS group; ^§^ *p* < 0.05 vs. DOX group.

Both free doxorubicin and doxorubicin-loaded dendrimersomes have been shown to be efficiently accumulated in 1301 cells (Figures S3 and S4). However, treating cells with free doxorubicin (3 µM) resulted in slight increasing of early and late apoptosis cell fractions in comparison with control (ca. 10% each), whereas doxorubicin-loaded dendrimersomes (3 µM doxorubicin) caused a sharp increase of late apoptosis and necrosis cell fractions (50% and 20%, respectively) (Figure 6 and Figure S5). Blank dendrimersomes do not cause any significant effect on the 1301 cells in comparison to control at the concentration used (5 µM dendron). Certain differences with WST data likely occurred due to methodological features of the techniques.

Our findings show that the encapsulation does not improve the rate of accumulation of doxorubicin into tumour cells, however, the cytotoxic effect of the doxorubicin-containing nanoconstruction is visibly higher. This likely occurs due to the switch of the mechanism of drug penetration into a cell. Remarkably, the presence of serum, an important component of the cell culture medium, does not suppress the dendrimersomes’ penetration into cells nor their cytostatic activity. The interaction of nanoconstructions with serum components is known to imminently take place during co-incubation with cells. We suppose that the encapsulation of anti-cancer chemodrugs into dendrimersomes can prolongate their persistence in the organism as well as reduce side effects. Such a phenomenon has been reported while using supramolecular associates [37] and macromolecules, in particular, dendrimers [38,39], as carriers for chemodrugs. In respect of novel amphiphilic triazine-carbosilane dendrimersomes reported herein, this hypothesis deserves further validation in animal experiments.

## 4. Conclusions

In summary, we have designed a new class of dendritic amphiphiles self-assembling into vesicle-like nanosized supramolecular associates (dendrimersomes). Rationally designed molecular topology of dendrons permits to use simple procedures to yield monodisperse nanoparticles. Because the reorganization is driven by subtle changes of ambient pH, triazine-carbosilane dendrimersomes can be useful as carriers for the delivery of sensitive, poorly soluble or highly toxic compounds into cells. As a proof-of-concept study, we attempted to encapsulate anti-cancer drugs doxorubicin and methotrexate and to deliver them into human leukaemia cells. The encapsulation, though efficient, does not change the vesicular topology of nanoconstructions. Drug-loaded dendrimersomes efficiently penetrate into cells and induce cell death. Our findings suggest that triazine-carbosilane dendrimersomes hold considerable potential for nanomedicine as stimuli-sensitive drug carriers.

## Figures and Tables

**Table 1:** Properties of drug-loaded dendrimersomes.

Sample ^1^	Particle Size ^2^, nm	PDI	Zeta Potential ^2^, mV	Drug Encapsulation ^2^, %	Drug Loading Capacity ^3^, %
DOX@DS	92 ± 38	0.213	41.3 ± 7.3	65 ± 10	>20.0
MTX@DS	46 ± 17	0.292	1.2 ± 4.5	75 ± 10	>20.0
5FU@DS	75 ± 30	0.216	11.0 ± 5.7	25 ± 5	>2.1

^1^ DS-dendrimersomes; ^2^ Mean ± S.D.; ^3^ rough estimation, see Section 2.8.

**Table 2:** IC_50_ values of blank dendrimersomes, chemodrugs and drug-loaded dendrimersomes towards 1301 and K562 cells.

Sample ^1^	1301	K562
DS	5.4 ± 1.4 µM	4.6 ± 2.6 µM
DOX	0.059 ± 0.021 µM ^2^	0.20 ± 0.08 µM
DOX@DS	0.71 ± 0.13 µM	1.2 ± 0.6 µM
MTX	0.99 ± 0.34 µM	0.046 ± 0.03 µM ^2^
MTX@DS	0.99 ± 0.40 µM	0.15 ± 0.08 µM

^1^ DS-dendrimersomes; ^2^ value predicted from data fitting

## Supplementary Materials

The following are available online at https://www.mdpi.com/2079-4991/10/10/1899/s1, Figure S1: Kinetic profiles of the doxorubicin release; Figure S2: Dendron biocompatibility; Figure S3: Flow cytometry plots; Figure S4: Fluorescence microscopy images; Figure S5: Flow cytometry plots.

## References

[1] Jain K., Mehra N.K., Jain N.K. (2014). Potentials and emerging trends in nanopharmacology. Curr. Opin. Pharmacol..

[2] Li Z., Ye E., David, Lakshminarayanan R., Loh X.J. (2016). Recent Advances of using hybrid nanocarriers in remotely controlled therapeutic delivery. Small.

[3] Movassaghian S., Merkel O.M., Torchilin V.P. (2015). Applications of polymer micelles for imaging and drug delivery. Wiley Interdiscip. Rev. Nanomed. Nanobiotechnol..

[4] Dzmitruk V., Apartsin E., Ihnatsyeu-Kachan A., Abashkin V., Shcharbin D., Bryszewska M. (2018). Dendrimers show promise for sirna and microrna therapeutics. Pharmaceutics.

[5] Knauer N., Pashkina E., Apartsin E. (2019). Topological aspects of the design of nanocarriers for therapeutic peptides and proteins. Pharmaceutics.

[6] Palmerston Mendes L., Pan J., Torchilin V. (2017). Dendrimers as nanocarriers for nucleic acid and drug delivery in cancer therapy. Molecules.

[7] Bolu B., Sanyal R., Sanyal A. (2018). Drug delivery systems from self-assembly of dendron-polymer conjugates. Molecules.

[8] Sandoval-Yañez C., Castro Rodriguez C. (2020). Dendrimers: Amazing Platforms for Bioactive Molecule Delivery Systems. Materials.

[9] Fernandez J., Acosta G., Pulido D., Malý M., Copa-Patiño J.L., Soliveri J., Royo M., Gómez R., Albericio F., Ortega P. (2019). Carbosilane dendron–peptide nanoconjugates as antimicrobial agents. Mol. Pharm..

[10] Sepúlveda-Crespo D., de la Mata F.J., Gómez R., Muñoz-Fernández M.A. (2018). Sulfonate-ended carbosilane dendrimers with a flexible scaffold cause inactivation of HIV-1 virions and gp120 shedding. Nanoscale.

[11] Krasheninina O., Apartsin E., Fuentes E., Szulc A., Ionov M., Venyaminova A., Shcharbin D., De la Mata F., Bryszewska M., Gόmez R. (2019). Complexes of pro-apoptotic siRNAs and carbosilane dendrimers: Formation and effect on cancer cells. Pharmaceutics.

[12] Sánchez-Milla M., Muñoz-Moreno L., Sánchez-Nieves J., Malý M., Gómez R., Carmena M.J., de la Mata F.J. (2019). Anticancer activity of dendriplexes against advanced prostate cancer from protumoral peptides and cationic carbosilane dendrimers. Biomacromolecules.

[13] Carloni R., Sanz del Olmo N., Ortega P., Fattori A., Gómez R., Ottaviani M.F., García-Gallego S., Cangiotti M., de la Mata F.J. (2019). Exploring the interactions of ruthenium (ii) carbosilane metallodendrimers and precursors with model cell membranes through a dual spin-label spin-probe technique using EPR. Biomolecules.

[14] Gutierrez-Ulloa C.E., Buyanova M.Y., Apartsin E.K., Venyaminova A.G., de la Mata F.J., Gómez R. (2018). Carbon nanotubes decorated with cationic carbosilane dendrons and their hybrids with nucleic acids. ChemNanoMat.

[15] Pędziwiatr-Werbicka E., Gorzkiewicz M., Horodecka K., Abashkin V., Klajnert-Maculewicz B., Peña-González C.E., Sánchez-Nieves J., Gómez R., de la Mata F.J., Bryszewska M. (2020). Silver nanoparticles surface-modified with carbosilane dendrons as carriers of anticancer siRNA. Int. J. Mol. Sci..

[16] Percec V., Wilson D.A., Leowanawat P., Wilson C.J., Hughes A.D., Kaucher M.S., Hammer D.A., Levine D.H., Kim A.J., Bates F.S. (2010). Self-Assembly of janus dendrimers into uniform dendrimersomes and other complex architectures. Science.

[17] Peterca M., Percec V., Leowanawat P., Bertin A. (2011). Predicting the size and properties of dendrimersomes from the lamellar structure of their amphiphilic janus dendrimers. J. Am. Chem. Soc..

[18] Thota B.N.S., Berlepsch H.v., Böttcher C., Haag R. (2015). Towards engineering of self-assembled nanostructures using non-ionic dendritic amphiphiles. Chem. Commun..

[19] Wei T., Chen C., Liu J., Liu C., Posocco P., Liu X., Cheng Q., Huo S., Liang Z., Fermeglia M. (2015). Anticancer drug nanomicelles formed by self-assembling amphiphilic dendrimer to combat cancer drug resistance. Proc. Natl. Acad. Sci. USA.

[20] Liu X., Zhou J., Yu T., Chen C., Cheng Q., Sengupta K., Huang Y., Li H., Liu C., Wang Y. (2014). Adaptive amphiphilic dendrimer-based nanoassemblies as robust and versatile siRNA delivery systems. Angew. Chem. Int. Ed..

[21] Gutierrez-Ulloa C.E., Buyanova M.Y., Apartsin E.K., Venyaminova A.G., de la Mata F.J., Valiente M., Gómez R. (2017). Amphiphilic carbosilane dendrons as a novel synthetic platform toward micelle formation. Org. Biomol. Chem.

[22] Mencia G., Lozano-Cruz T., Valiente M., de la Mata J., Cano J., Gómez R. (2020). New ionic carbosilane dendrons possessing fluorinated tails at different locations on the skeleton. Molecules.

[23] Gutierrez-Ulloa C.E., Sepúlveda-Crespo D., García-Broncano P., Malý M., Muñoz-Fernández M.A., de la Mata F.J., Gómez R. (2019). Synthesis of bow-tie carbosilane dendrimers and their HIV antiviral capacity: A comparison of the dendritic topology on the biological process. Eur. Polym. J..

[24] Moreno K.X., Simanek E.E. (2008). Identification of diamine linkers with differing reactivity and their application in the synthesis of melamine dendrimers. Tetrahedron Lett..

[25] Lim J., Simanek E.E. (2012). Triazine dendrimers as drug delivery systems: From synthesis to therapy. Adv. Drug Deliv. Rev..

[26] Bagul R.S., Hosseini M.M., Shiao T.C., Roy R. (2017). “Onion peel” glycodendrimer syntheses using mixed triazine and cyclotriphosphazene scaffolds. Can. J. Chem..

[27] Ji K., Lee C., Janesko B.G., Simanek E.E. (2015). Triazine-Substituted and acyl hydrazones: Experiment and computation reveal a stability inversion at low pH. Mol. Pharm..

[28] Poletaeva J., Dovydenko I., Epanchintseva A., Korchagina K., Pyshnyi D., Apartsin E., Ryabchikova E., Pyshnaya I. (2018). Non-Covalent associates of siRNAs and AuNPs enveloped with lipid layer and doped with amphiphilic peptide for efficient siRNA delivery. Int. J. Mol. Sci..

[29] Onciu M. (2009). Acute lymphoblastic leukemia. Hematol. Oncol. Clin. N. Am..

[30] Saußele S., Silver R.T. (2015). Management of chronic myeloid leukemia in blast crisis. Ann. Hematol..

[31] Fuentes-Paniagua E., Peña-González C.E., Galán M., Gómez R., De La Mata F.J., Sánchez-Nieves J. (2013). Thiol-ene synthesis of cationic carbosilane dendrons: A new family of synthons. Organometallics.

[32] Aguiar J., Carpena P., Molina-Bolívar J.A., Carnero Ruiz C. (2003). On the determination of the critical micelle concentration by the pyrene 1:3 ratio method. J. Colloid Interface Sci..

[33] Sánchez-Nieves J., Ortega P., Muñoz-Fernández M.Á., Gómez R., de la Mata F.J. (2010). Synthesis of carbosilane dendrons and dendrimers derived from 1,3,5-trihydroxybenzene. Tetrahedron.

[34] Mooibroek T.J., Gamez P. (2007). The s-triazine ring, a remarkable unit to generate supramolecular interactions. Inorganica Chim. Acta.

[35] Parkar N.S., Akpa B.S., Nitsche L.C., Wedgewood L.E., Place A.T., Sverdlov M.S., Chaga O., Minshall R.D. (2009). Vesicle formation and endocytosis: Function, machinery, mechanisms, and modeling. Antioxid. Redox Signal..

[36] Klein E., Vánky F., Ben-Bassat H., Neumann H., Ralph P., Zeuthen J., Polliack A. (1976). Properties of the K562 cell line, derived from a patient with chronic myeloid leukemia. Int. J. Cancer.

[37] Fahmy S.A., Brüßler J., Alawak M., El-Sayed M.M.H., Bakowsky U., Shoeib T. (2019). Chemotherapy based on supramolecular chemistry: A promising strategy in cancer therapy. Pharmaceutics.

[38] Abedi-Gaballu F., Dehghan G., Ghaffari M., Yekta R., Abbaspour-Ravasjani S., Baradaran B., Ezzati Nazhad Dolatabadi J., Hamblin M.R. (2018). PAMAM dendrimers as efficient drug and gene delivery nanosystems for cancer therapy. Appl. Mater. Today.

[39] Hossain Sk U., Kojima C., Rahman A. (2015). Dendrimers for Drug Delivery of Anticancer Drugs. Frontiers in Clinical Drug Research-Anti-Cancer Agents.

