# Supplementary material for: pH-Sensitive Dendrimersomes of Hybrid Triazine-Carbosilane Dendritic Amphiphiles-Smart Vehicles for Drug Delivery
Source: Nanomaterials (Basel). 2020 Sep 23;10(10):1899. doi: 10.3390/nano10101899 (PMC7598245; doi:10.3390/nano10101899)

nanomaterials

Supplementary Materials

pH-Sensitive Dendrimersomes of Hybrid Triazine-Carbosilane Dendritic Amphiphiles-Smart Vehicles for Drug Delivery

Evgeny Apartsin [1,2,3,*], Nadezhda Knauer [1,4], Valeria Arkhipova [1,2], Ekaterina Pashkina [1,4], Alina Aktanova [1,4], Julia Poletaeva [1], Javier Sánchez-Nieves [5,6], Francisco Javier de la Mata [5,6,7] and Rafael Gómez [5,6,7,*]

1. Institute of Chemical Biology and Fundamental Medicine SB RAS, 8, Lavrentiev ave., 630090 Novosibirsk, Russia; knauern@gmail.com (N.K.); v.arkhipova@g.nsu.ru (V.A.); pashkina.e.a@yandex.ru (E.P.); aktanova_al@mail.ru (A.A.); fabaceae@yandex.ru (J.P.)
2. Department of Natural Sciences, Novosibirsk State University, 630090 Novosibirsk, Russia
3. Laboratoire de Chimie de Coordination, CNRS, 31077 Toulouse, France
4. Research Institute of Fundamental and Clinical Immunology, 630099 Novosibirsk, Russia; knauern@gmail.com (N.K.), pashkina.e.a@yandex.ru (E.P.), aktanova_al@mail.ru (A.A.)
5. Departamento de Química Orgánica y Química Inorgánica, UAH-IQAR, Universidad de Alcalá, 28805 Alcalá de Henares, Spain; javier.sancheznieves@uah.es (J.S.-N.), javier.delamata@uah.es (F.J.d.l.M.)
6. Networking Research Center on Bioengineering, Biomaterials and Nanomedicine (CIBER-BBN), Madrid, Spain
7. Instituto Ramón y Cajal de Investigación Sanitaria, IRYCIS, Madrid, Spain

* Correspondence: eka@niboch.nsc.ru (E.A.); rafael.gomez@uah.es (R.G.)

DOX release from dendrimersomes

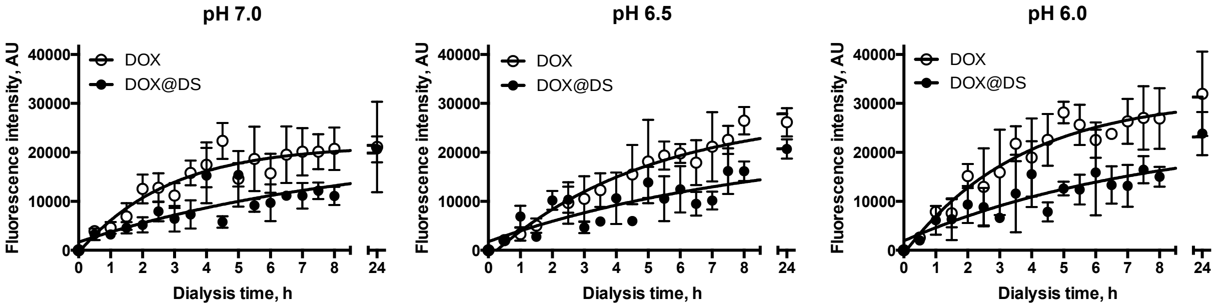

Figure S1. Kinetic curves of the fluorescence acquisition in the dialysis buffer upon incubation of free doxorubicin and doxorubicin-loaded dendrimersomes at different pH. Conditions: see Section 2.9.

Dendron biocompatibility

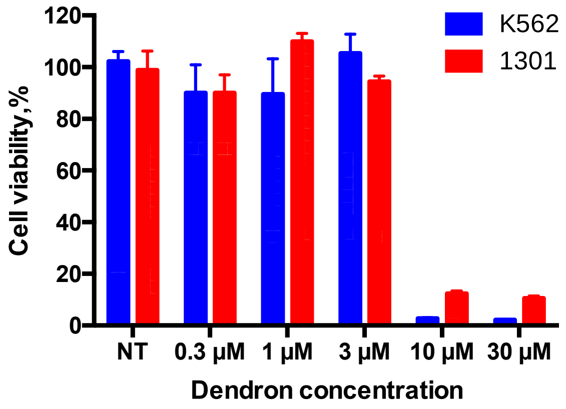

Figure S2. Cell viability profiles after incubation with amphiphilic dendron for 72 h. WST-1 assay. NT–non-treated cells. Data are presented as Mean ± S.D. (n = 5).

Drug internalization studies

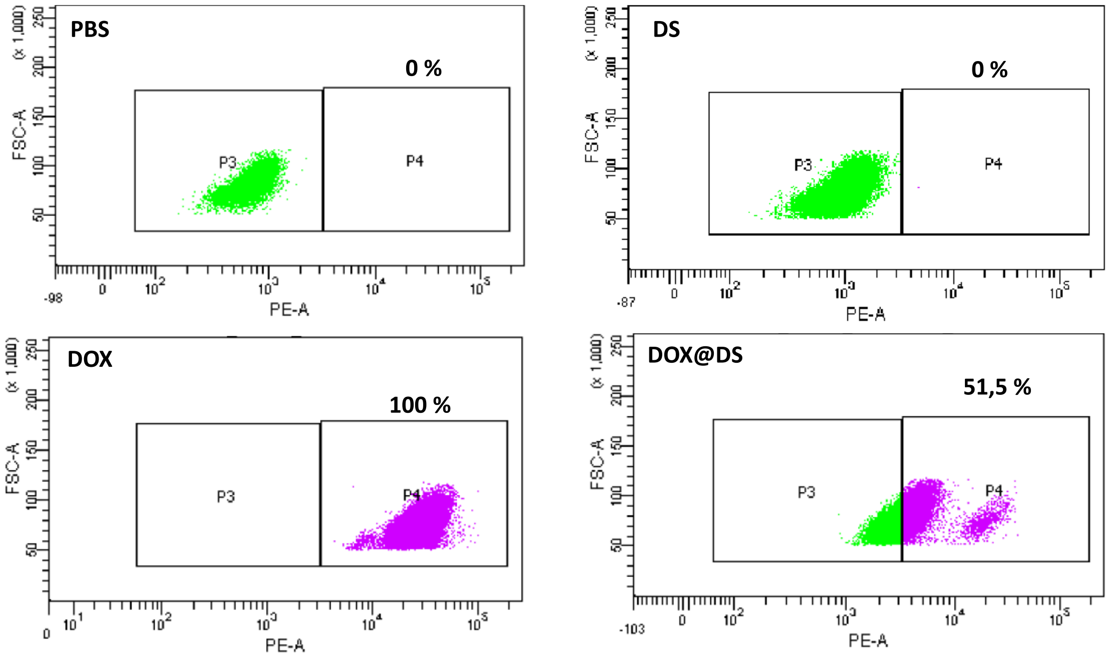

Figure S3. Representative plots of fluorescence distribution in 1301 cells incubated with PBS (non-treated control), blanc dendrimersomes (DS), doxorubicin (DOX) and doxorubicin-loaded dendrimersomes (DOX@DS). Percentages of cells having fluorescence higher than in control are indicated on graphs.

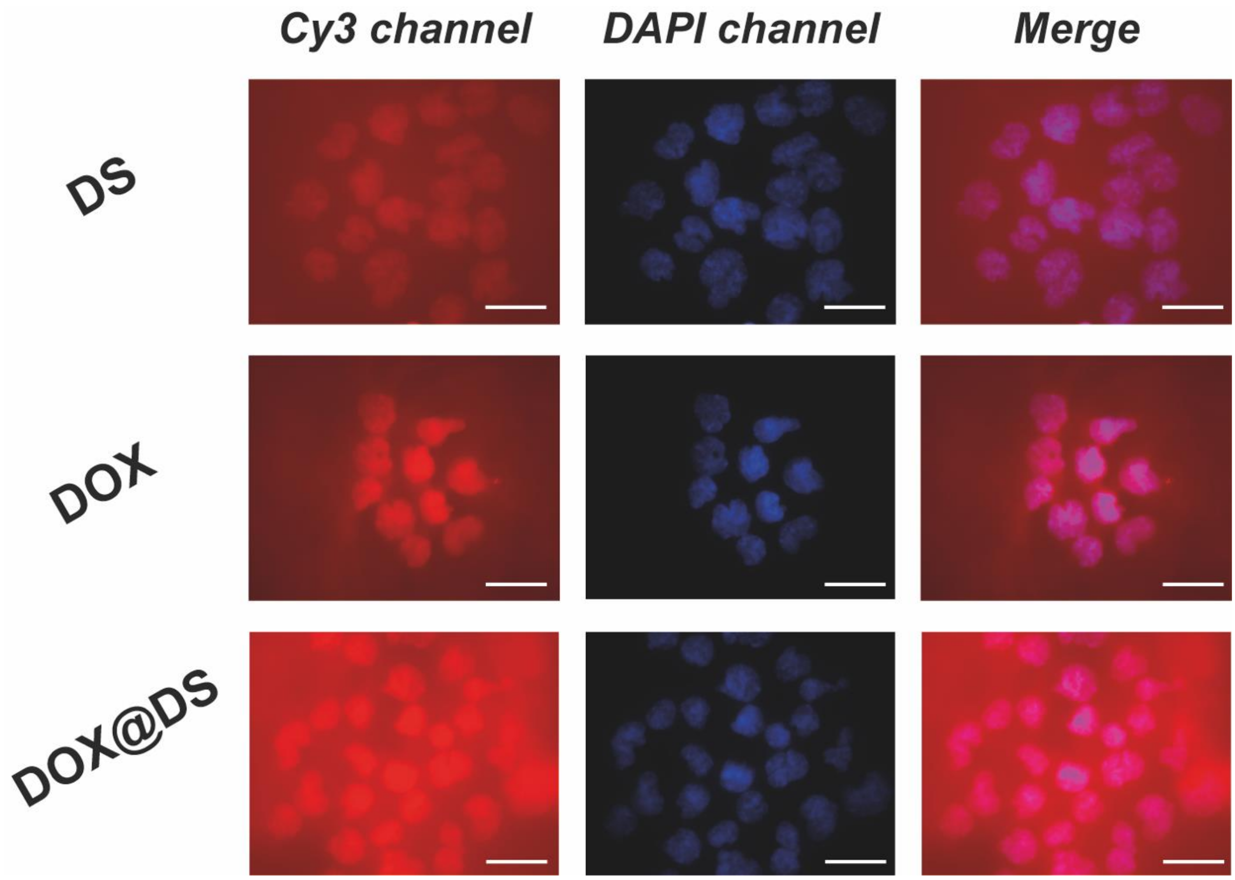

Figure S4. Fluorescence microscopy images of 1301 cells incubated with blanc dendrimersomes (DS), doxorubicin (DOX) and doxorubicin-loaded dendrimersomes (DOX@DS). Scale bar represents 20 µm.

Apoptosis induction studies

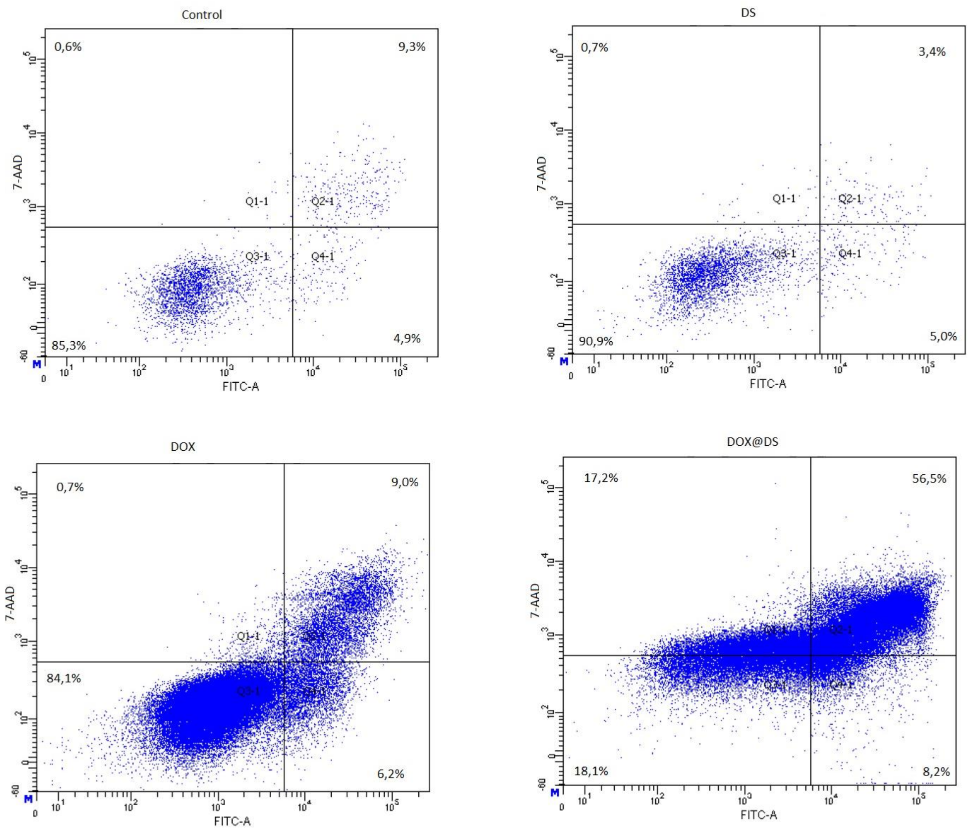

Figure S5. Representative FACS plots of 1301 cells incubated with PBS (non-treated control), blanc dendrimersomes (DS), doxorubicin (DOX) and doxorubicin-loaded dendrimersomes (DOX@DS) followed by staininig with FITC-Annexin V and 7-AAD.

1. H NMR spectra ($CDCl_3$)

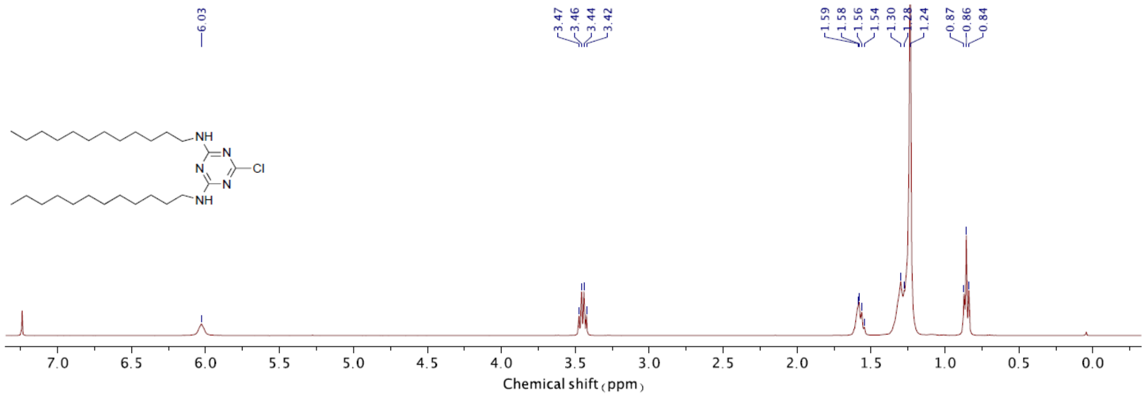

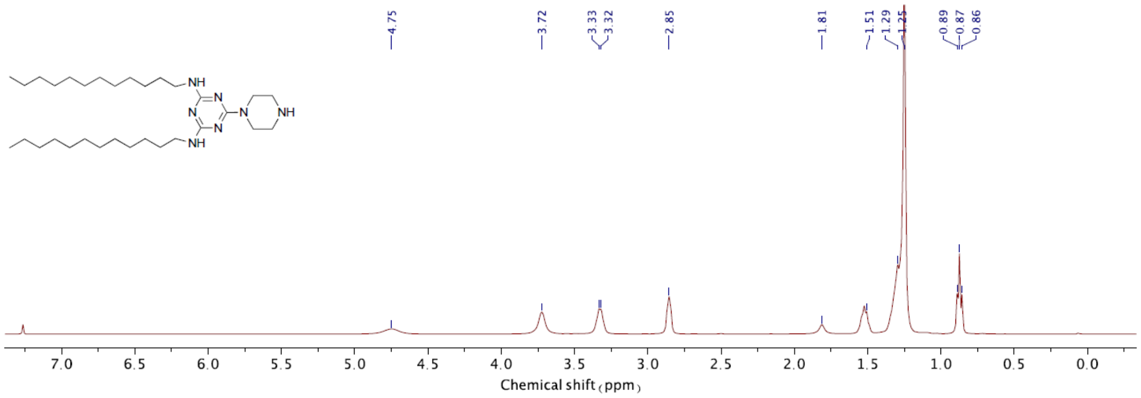

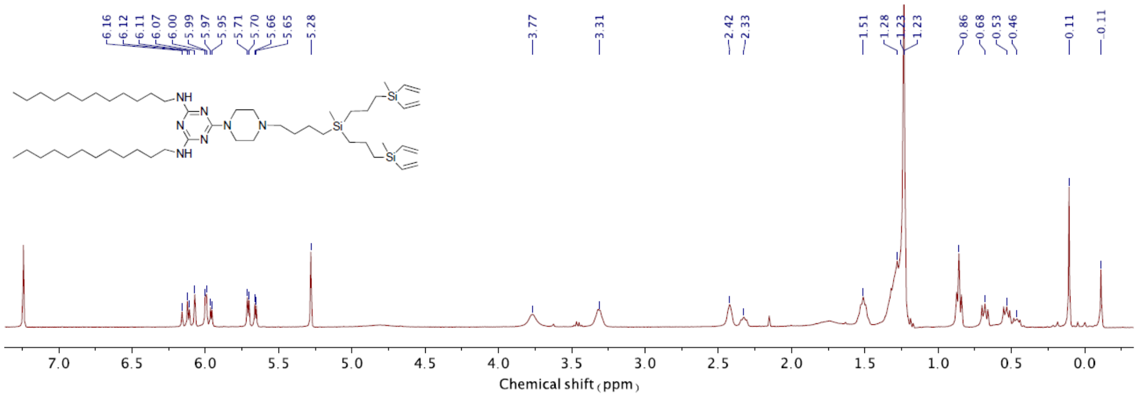

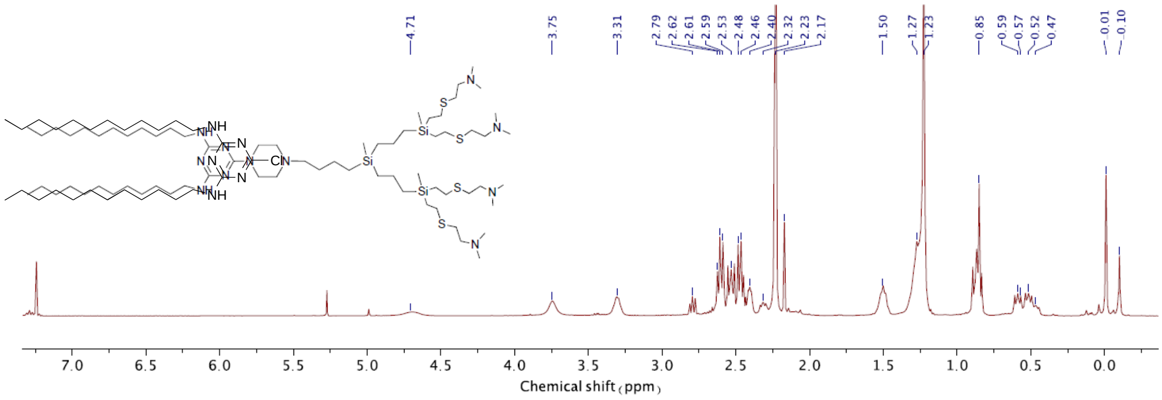

MS spectrum of the amphiphilic dendron G2

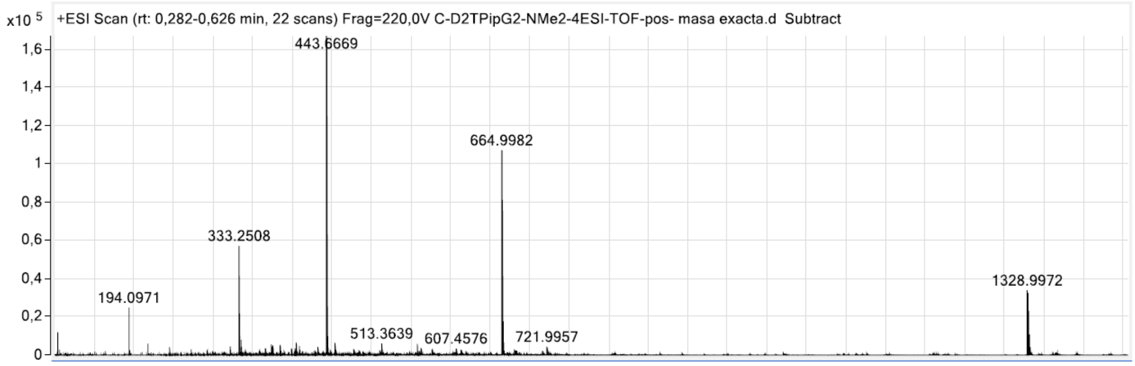

Supplement: Supplementary file 1 [file nanomaterials-10-01899-s001.pdf]
